# Comparison of ultrasound-guided modified Seldinger technique versus blind puncture for peripherally inserted central catheter: a meta-analysis of randomized controlled trials

**DOI:** 10.1186/s13054-015-0742-y

**Published:** 2015-02-14

**Authors:** ZhanZhan Li, LiZhang Chen

**Affiliations:** Department of Epidemiology and Health Statistics, School of Public Health, Central South University, Xiangya Road, Kaifu District, Changsha, Hunan Province 410078 China

The peripherally inserted central catheter (PICC) has been used for central venous pressure monitoring and building effective infusion routes in a critical care setting. The traditional blind puncture technique for PICC has been challenging. The ultrasound-guided modified Seldinger technique (MST) has been used as an adjunct for central venous catheter and has shown significant benefits over the traditional blind puncture. However, despite clear advantages, complications of ultrasound-guided MST for PICC still occur. We conducted a meta-analysis of randomized controlled trials (RCTs) to compare the ultrasound-guided MST and traditional blind puncture techniques for PICC.

PubMed and CNKI (Chinese database) were searched for RCTs comparing the ultrasound-guided MST and traditional palpation for radial artery catheterization. Differences were expressed as relative risks (RRs) with 95% confidence intervals (CIs) for dichotomous outcomes. The fixed-effects model or random-effects model was used, depending on whether heterogeneity existed among studies. Heterogeneity among studies was examined with Cochran’s *Q* statistic (*P* < 0.1) and the *I*^*2*^ statistic. A two-tailed *P* value of less than 0.05 was considered a significant level except for where a certain *P* value has been given. Six RCTs enrolling 726 patients (367 trials and 359 controls) were included in the meta-analysis [1-6]. Three studies [1,3,4] report that the first-attempt success rate of ultrasound-guided MST was superior to that of the traditional blind puncture, and three studies [2,5,6] report that the first-attempt success rate was similar for the two techniques. The meta-analysis indicated that ultrasound-guided MST was not significantly associated with improvement in first-attempt success (RR = 1.07, 95% CI 0.99 to 1.16, *P* = 0.090) compared with the traditional blind puncture, but ultrasound-guided MST significantly reduced the incidence of complication after surgery (RR = 0.24, 95% CI 0.08 to 0.76, *P* = 0.015).

In summary, ultrasound-guided MST is superior to the traditional blind puncture technique for PICC, especially for postoperative complications. It should be generalized to clinical application for PICC.

## Abbreviations

CI: Confidence interval
MST: Modified Seldinger technique
PICC: Peripherally inserted central catheter
RCT: Randomized controlled trial
RR: Relative risk
